# Alternative initiation regimen of paliperidone palmitate long-acting injectable

**DOI:** 10.1192/j.eurpsy.2022.728

**Published:** 2022-09-01

**Authors:** I.E. Menendez Gil, S.L. Romero Guillena, B.O. Plasencia Garcia De Diego

**Affiliations:** 1 Virgen del Rocio Hospital, Psychiatry, Seville, Spain; 2 UGC Salud Mental Virgen Macarena,, Psychiatry, seville, Spain

**Keywords:** schizophrénia, paliperidone palmitate long-acting, antipsychotic, Psychosis

## Abstract

**Introduction:**

Long-acting injectable antipsychotics (LAIs) hold an important place in the treatment of psychosis. Knowledge of the best way to administer LAIs is important to maximize the efficacy and minimize the side-effects

**Objectives:**

To assess the effectiveness of flexible doses of palmitate paliperidone long-acting injectable (PPLAI) against standard doses in initiation regimen in the subset of acutely hospitalized patients with schizophrenia and other psychosis

**Methods:**

Retrospective, noninterventional study. Group of initiation regimen: A)“Standard-doses” (recommended PPLAI initiation regimen: 150mg-Day-1 and 100mg-Day-8±4days), B)“Low-doses” (any dosage lower than the Standard-dose) C)“High-doses” (150mg-Day-1 and 150mg-Day-8±4days) Effectiveness was measured with the number of psychiatric hospital admission and psychiatric emergency visit 6-months post-discharge. Length of stay of the index hospitalization, adherence to treatment and adverse events was confirmed in the medical record. Concomitant use of biperiden was recorded.

**Results:**

51 patients were included. We found no statistically differences in study variables between groups (Table-1). Table-1.

**Table:** 

	Standard doses(n=31)	Low-doses(n=13)	High-doses(n=7)
Length of stay mean±sd	17.23±13.09	13.77±9.02	17±10.55
No psychiatric hospitalizations 6-month post-discharge, %patients( n)	71%(n=22)	84.6%(n=11)	85.7%(n=6)
No psychiatric emergency visits 6-month post-discharge, %patients(n)	61.3%(n=19)	69.2%(n=9)	85.7%(n=6)
Prescription of biperiden 6-month post-discharge, %patients(n)	13.3%(n=4)	0%(n=0)	14.3%(n=1)
Adherence to treatment 6-month post-discharge, %patients(n)	80.6%(n=25)	84.61%(n=11)	57.1%(n=4)

**Conclusions:**

No differences were found in the effectiveness of flexible-doses in PPLAI initiation regimen. The use of low doses of PPLAI could keep the efficacy of the standard dose with a better side effect profile and treatment adherence. The strength of the conclusion is limited by the design and the number of patients.

**Disclosure:**

No significant relationships.

